# Information-Theoretic Modeling of Categorical Spatiotemporal GIS Data

**DOI:** 10.3390/e26090784

**Published:** 2024-09-13

**Authors:** David Percy, Martin Zwick

**Affiliations:** 1 Geology Department, Portland State University, Portland, OR 97207, USA; 2 Complex Systems Department, Portland State University, Portland, OR 97207, USA; zwick@pdx.edu

**Keywords:** GIS, reconstructability analysis, spatiotemporal, categorical data, forest prediction, space–time data cube

## Abstract

An information-theoretic data mining method is employed to analyze categorical spatiotemporal Geographic Information System land use data. Reconstructability Analysis (RA) is a maximum-entropy-based data modeling methodology that works exclusively with discrete data such as those in the National Land Cover Database (NLCD). The NLCD is organized into a spatial (raster) grid and data are available in a consistent format for every five years from 2001 to 2021. An NLCD tool reports how much change occurred for each category of land use; for the study area examined, the most dynamic class is Evergreen Forest (EFO), so the presence or absence of EFO in 2021 was chosen as the dependent variable that our data modeling attempts to predict. RA predicts the outcome with approximately 80% accuracy using a sparse set of cells from a spacetime data cube consisting of neighboring lagged-time cells. When the predicting cells are all Shrubs and Grasses, there is a high probability for a 2021 state of EFO, while when the predicting cells are all EFO, there is a high probability that the 2021 state will not be EFO. These findings are interpreted as detecting forest clear-cut cycles that show up in the data and explain why this class is so dynamic. This study introduces a new approach to analyzing GIS categorical data and expands the range of applications that this entropy-based methodology can successfully model.

## 1. Introduction

**Figure 1:**
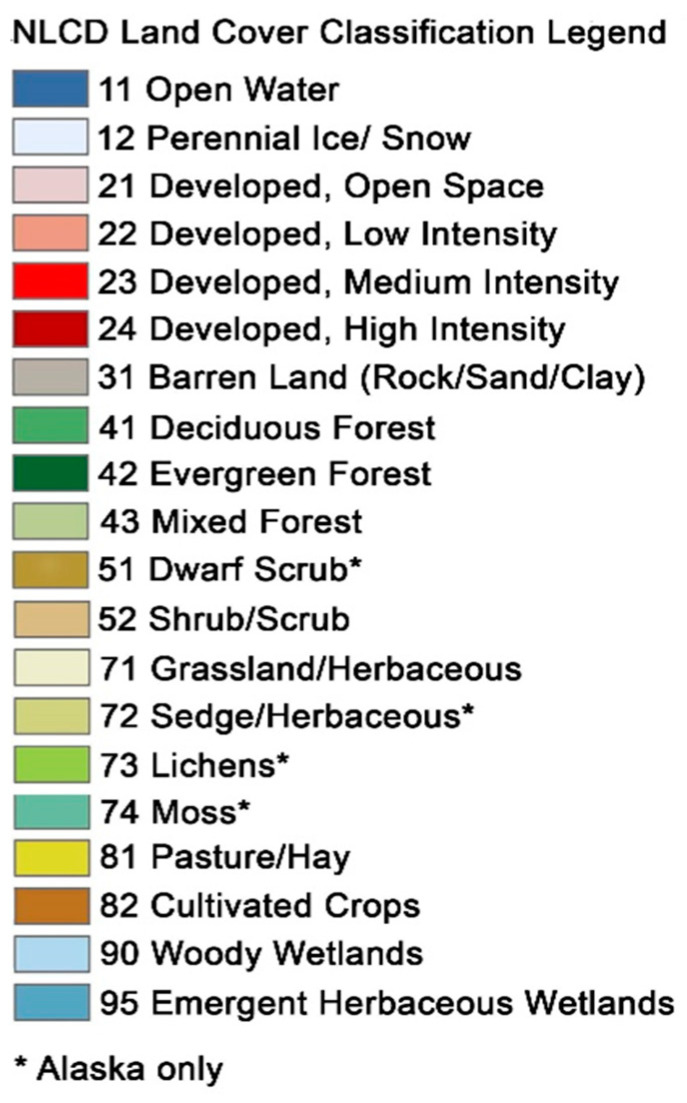
Legend for National Land Cover Database (NLCD) showing the 20 classes and the numeric coding for each. As noted in the graphic, four classes are only available in Alaska. Perennial ice did not appear in our data, so we have 15 classes. For this study, the categories were organized into five final classes: Water, Developed, and Agriculture (WDA), Shrubs (SHBs), Grasses (GRSs), Mixed Forest (MFO), and Evergreen Forest (EFO).

**Figure 2:**
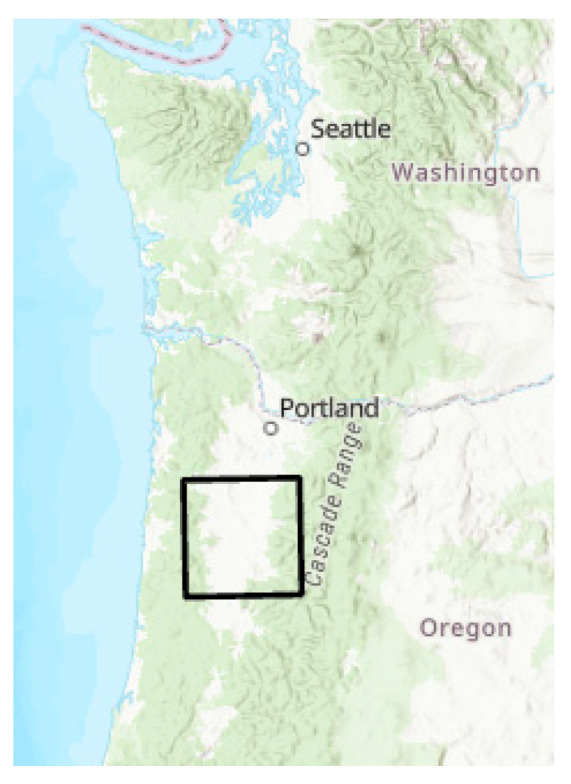
Study area. Black outline shows the location of the study area for this project, in the Willamette Valley of Oregon, West Coast, USA.

Land cover data are generated using satellites and processed into broad classes that capture general land usage [1], as shown in Figure 1. For the time period of 2001 to 2021, the US-funded National Land Cover Database (NLCD) project has produced a well-organized and formatted data set for the entire US collected every two to three years [2]. We extracted from this data set an area of interest in the Willamette Valley of the state of Oregon, a diverse landscape near where the researchers live (Figure 2).

Using Reconstructability Analysis (RA), as implemented in OCCAM software (Version 3.4) [3], we analyze these data to test our ability to predict (reduce the Shannon entropy of) future states based on past information [4]. RA is used to find constraint among discrete variables in a manner similar to the way that multiple linear regression finds a correlation among continuous variables. RA searches for good models of the data, one of which is then used to make explicit predictions of the DV (dependent variable) from the IVs (independent variables). These are two separate functions in OCCAM: its “Search” function looks for good models, i.e., models that reduce the entropy of the DV and are not overly complex; its “Fit” function generates predictions from the data (probability distributions for the DV conditioned on the IV state) for the model that the user has selected [3].

**Figure 3:**
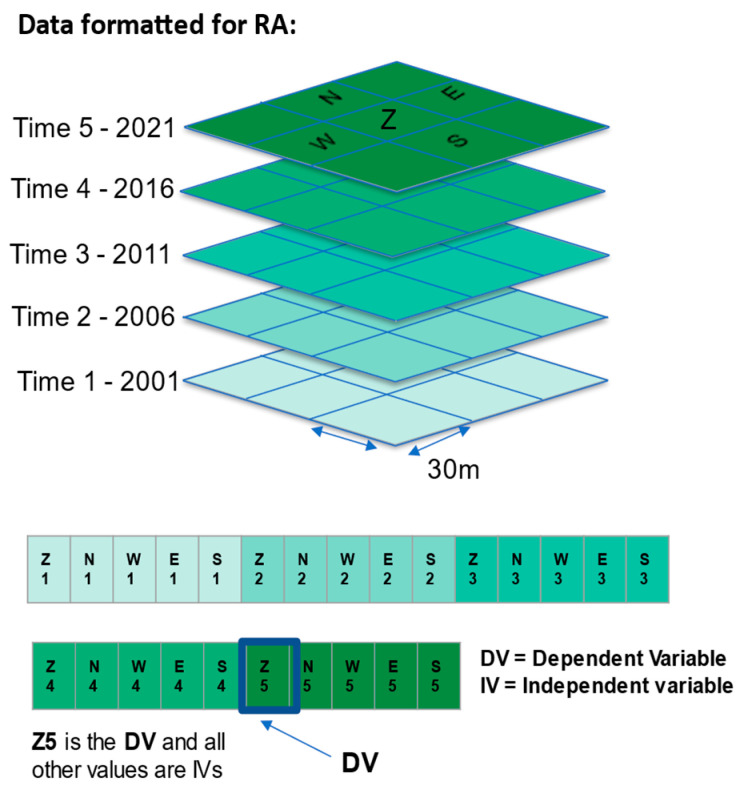
Space–time data cube with 30 m grid cells extracted to a row of data for Reconstructability Analysis. Cells are named with their cardinal direction plus the time number. The dependent variable, DV, is the center cell of the most recent year.

Raster data, like the NLCD, are structured in a rectangular grid with one value per cell. When these grids are stacked, with one layer per time slice, it becomes a space–time data cube (Figure 3). We attempt to predict the state of the center cell of the most current year (2021, referred to as time 5) on the basis of information on past states of the center cell and its neighborhood cells in the four cardinal directions. The past states are 2001, 2006, 2011, and 2016 and are referred to as times 1 through 4. Previous years of NLCD data are available, but they are not uniformly organized the way that the 2001 to 2021 data are.

**Figure 4:**
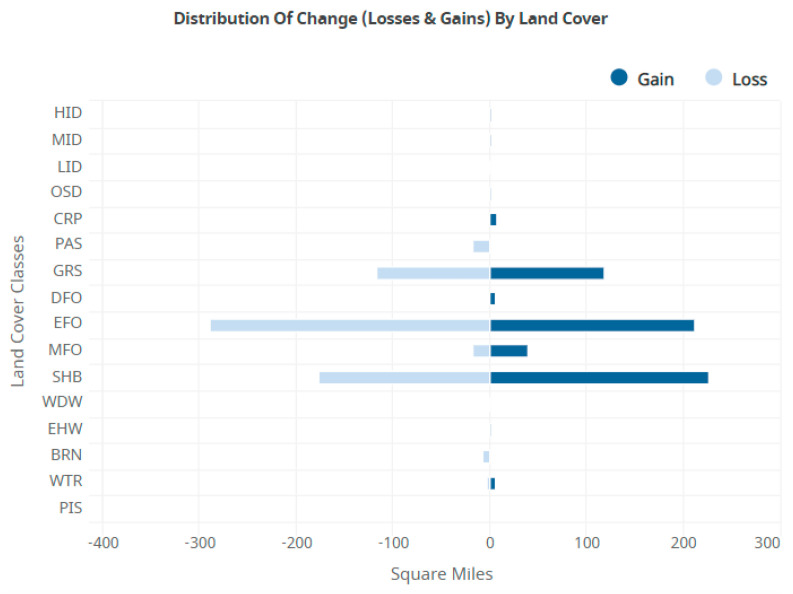
Report from the NLCD website for Lane County showing the gains and losses for each land use type. EFO (Evergreen Forest), GRS (grassland/herbaceous), and SHB (shrub/scrub) are the classes with the greatest amount of loss and gain.

OCCAM requires data in a tabular form with the cases in rows and the variables in columns, so we convert the entire neighborhood into a row of data. In this analysis, the center cell at time 5 in a 3 × 3 neighborhood is the DV. The states of this center cell plus four additional neighbor cells (north, west, east, and south) at the four previous years in the analysis are the IVs. More specifically, the presence/absence of Evergreen Forest at the center cell at time 5 is the actual DV we are trying to predict. We focus on this class because the analysis on the NLCD website indicates that Evergreen Forests were the most dynamic land cover type (Figure 4). We recode Z5 to be 42 (class EFO) vs. everything else. This two-state DV is used in an RA model with the IVs being the lagged states of the target cell plus their neighboring states, classified according to the NLCD. The 20 IV cells are named according to their cardinal directions: N, W, E, and S. The time variables are referred to by the numbers 1 through 4 for the 2001 to 2016 time-slices, so the north cell of 2001 is N1, while the south cell of 2006 is S2, etc. The lagged center cells from 2001 through 2016 are named Z1 through Z4.

There is a well-established research area known as Spatial Entropy [5,6] that uses entropy measures primarily to quantify the degree of diversity (disorder, uncertainty) within spatial data. The research presented in this paper uses entropy-based measures to analyze diachronic relations in GIS data between the state of sites (the DV) and the states of time-lagged neighborhood sites (IVs). This is related to the Spatial Entropy research area only in sharing a common mathematical foundation in Shannon’s information theory, i.e., the Spatial Entropy area does not encompass the kind of predictive spatial–temporal modeling reported in this paper. There are other entropy-related formalisms (e.g., Dempster–Shafer evidence theory) beyond Shannon’s classical framework that Klir [7] and other researchers have examined and developed. These other formalisms are especially relevant for uncertain and incomplete information. The NLCD data analyzed in this paper, however, are adequately modeled with the classical (Shannon information theory-based) RA methodology used in this project.

### 1.1. RA Background

Reconstructability Analysis (RA) is a technique for modeling data that is used to quantify the relationships in categorical data [4,8,9]. It can also be applied to continuous data if these data are discretized (binned). RA is rooted in Shannon’s Information Theory [10] and Ashby’s Constraint Analysis [11], with graphical modeling added by Klir, Krippendorff, and others [9,12]. RA calculates the amount of Information “transmitted” between discrete variables, resulting in a quantification of their relations. RA is a categorical technique that can be used in a way that is analogous to multiple linear regression analysis of continuous data. Reconstructability Analysis is a rich framework for analyzing and quantifying the relationships of parts to wholes, though in this project we use only a small portion of its capabilities.

We use a form of RA in which the data are treated as a directed system, where models are assessed by how much their predicting IVs reduce the Shannon entropy (uncertainty) of a DV. Let p(DV) be the observed probability distribution of the DV; let p_model_(DV|IV) be the calculated model conditional probability distribution of the DV given the predicting IVs in the model. Then, H(p(DV)) = −∑_i_ p(DV_i_) log_2_ p(DV_i_) is the Shannon entropy of the DV in the data and H(p_model_(DV|IV)) is the (smaller) Shannon entropy of the DV in the model. The difference between these two entropies is the DV entropy reduction achieved by the model and is the information-theoretic measure of the predictive efficacy of the IVs used in the model. Dividing this entropy reduction by H(p(DV)) gives the fractional entropy reduction, expressed in the OCCAM software as a percent.

The data are expected to have a tabular form, with each row representing an observation and each column representing a single variable with discrete values. One column is the DV and the other columns are the IVs. (In principle, RA could address multiple DVs, but the OCCAM software used in this research allows only a single DV for each analysis). This form of RA can determine not only which IVs are the best predictors of the DV (feature selection) but also to make specific predictions of the DV (derived from calculated conditional probabilities of the DV) given the composite IV state. The alternative to a directed system is a neutral system where no IV-DV distinction is made. For neutral systems, one is interested in all the relations that exist among all the variables; in effect, each variable is a DV and all other variables are IVs. Directed system analysis is much simpler than neutral system analysis because one selects only one variable as the DV. Another important distinction in RA modeling is between loopless models and models with loops. Loopless models have only one IV-DV relation that predicts the DV, while models with loops have multiple predictive IV-DV relations where these relations are fused together via a maximum entropy formalism to yield a net DV prediction from all of the predicting IVs. Models with loops are usually better predictors but they require iteration and thus longer computation times. In this study, we use models with loops because computation is fast enough to become a non-issue.

To illustrate the idea of a single predictive IV-DV relation, suppose we have three IVs called A, B, and C, and let us call the DV by the name Z. The observed data are a frequency distribution f(ABCZ) from which, normalizing by the sample size, the probability distribution p(ABCZ) is obtained. Here, p(DV) = p(Z), a projection of p(ABCZ). A predictive relation will include Z and some subset of {A, B, C}, prediction being based on the probability distribution of Z conditioned on this subset. For example, the best model with one predictor might have the predictive relation BZ, meaning that B predicts Z better than A and C, this relation predicting Z via the conditional distribution p(Z|B). The best no-loop model with two IV predictors might have the predictive relation be BCZ, where there is a three-way interaction effect between B, C, and Z. For models with loops, a two-predictor model might have the predictive relations be BZ:CZ, where B and C predict Z separately, but their predictions are fused via a maximum entropy formalism. These are the types of models you will see in this study. Note that in these models, the order of relations is irrelevant and the order of variables within any relation is also irrelevant, so that the previously mentioned model with loops could equally well have been written CZ:ZB, for example.

In addition to one or more predictive relations, directed systems models have an “IV component”, which is a relation among all and only the IVs, such as in the current example, ABC. This relation assures that all predictive models are hierarchically nested for statistical considerations; by lumping together all the IVs in a single relation, it abstains from positing any specific relations among different IVs, allowing the focus of the model to be on the predictive relations that include the DV. So, in the example given in the previous paragraph, the IV component would be ABC; the best loopless model would actually be ABC:BCZ. For this model,

p_model_(ABCZ) = p(ABC)p(BCZ)/p(BC) and

p_model_(DV|IV) = p_ABC:BCZ_(Z|BC). Including the IV component in the best with-loops model example gives ABC:BZ:CZ. The probability distribution for this with-loops model, p_ABC:BZ:CZ_(ABCZ), however, cannot be calculated algebraically but must be generated iteratively using the

IPF (iterated proportional fitting) algorithm or its equivalent. In this later model, the IV component causes the existence of the loop, which can be seen by replacing the IV component by the BC projection that it encompasses and writing the model as BC:CZ:ZB. By contrast, if one omitted the IV component, the BZ:CZ model does not have a loop, and its probability distribution can be calculated algebraically. Models of this sort are known as naïve-Bayes models and are not included in RA as implemented in OCCAM. For a discussion of the relation between RA and these naïve-Bayes models and other Bayesian network models, see Harris and Zwick, 2021 [13]. Finally, models are compared to a reference, typically the data (“top”), abbreviated simply as p, here p(ABCZ), or the independence (“bottom”) model, with distribution p_ind_ = p_ABC:Z_(ABCZ) = p(ABC)p(Z).

**Figure 5:**
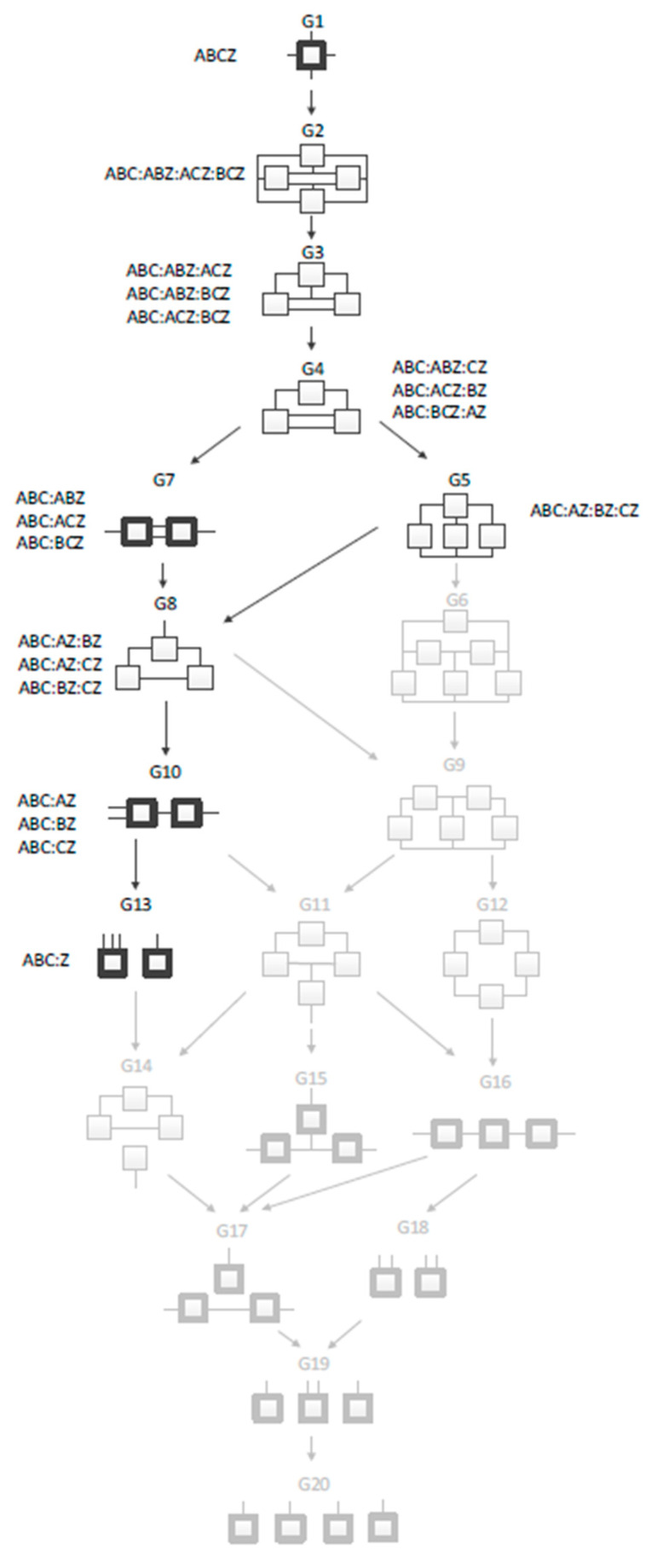
Lattice of Structures for a four-variable directed system (from [10]). Boxes represent relations, while lines indicate variables. Greyed out boxes indicate structures used only in neutral systems analysis. The four variables are fully connected at the top and completely independent at the bottom. Models with bold boxes do not have loops. Arrows indicate parent–child relationship between models.

RA uses hypergraphs as models, information-theoretic measures to quantify the goodness of models, and statistical methods to assess confidence in these measures. The set of candidate models defines a Lattice of Structures (hypergraphs) of all possible models for that many variables. Figure 5 shows an example of a four-variable system. The nine models not grayed out are the candidate models if the system is directed; the additional eleven grayed out models would need to be considered if the system were neutral. In these models, a box is a relation; a line (which may be branched) is a variable. The most complex possible model, at the top, is one with a single tetradic relation that fully connects all the variables. The least complex model, at the bottom, is one with a fully independent (disconnected) set of variables, which might be called monadic relations.

Intermediate models consist of multiple relations, each of which connects a subset of either three or two variables. Models without loops have relations shown in bold.

A candidate model is assessed by comparing it to a reference model, either the top (the data, the saturated model) or the bottom (the independence model). In this study, the bottom is the reference. Models are assessed based on how well they predict the DV using both information theoretic (entropy reduction) and a more generic machine learning metric (%Correct). Entropy reduction or normalized information captured in the model (1 for the data, 0 for the independence model), which is collinear with entropy reduction, when traded off against the added complexity of the candidate model beyond the independence model, helps to select a best model. Complexity is quantified as df, the degrees of freedom of the data or model distribution. Trade-off metrics in OCCAM are the Akaike and Bayesian Information Criteria (AIC, BIC) and Chi-square *p*-values.

The OCCAM implementation of RA, hosted on the servers at Portland State University, is divided into two primary functions, called “Search” and “Fit”. In Search, we supply a data file in a suitable format and receive as output a set of candidate models with metrics from which we choose a “best” model. The data and the chosen model are supplied to the Fit function, and a set of tables are returned which have every possible combination of states of the IVs and the conditional probabilities of DV states, given the IV states, from which the DV predictions are obtained for these IV states, along with a variety of metrics.

The discrete character of the NLCD suggests RA as a potentially valuable analysis technique. NLCD data, for the purposes of this study, define 15 classes of land use types (Figure 1). Since these data are classified in a format readily amenable to RA, we decided to analyze them with OCCAM to see what predictive relations would emerge. For this study, we reclassify the data to five classes, which are Water, Developed, and Agriculture (1), Grasses (2), Shrubs (3), Mixed and Deciduous Forest (4), and Evergreen Forest (5). This reduction in the cardinality of the variables makes post-processing analysis more manageable, as we will see in the results section. The data are analyzed for five years: 2001, 2006, 2011, 2016, and 2021, referred to, respectively, as times 1 through 5. NLCD data are reported in a grid of 30 m cell size, with an accuracy of around 80% (USGS), having been classified from satellite images.

### 1.2. Previous Work

**Figure 6:**
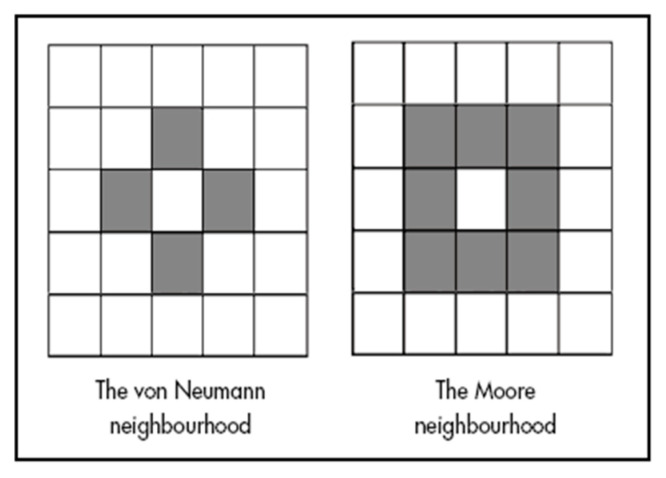
Two possible spatial neighborhoods using either 4 or 8 cells. The 4-neighbor von Neumann (VNN) is used in this study. Other spatial arrangements are possible, such as triangles or hexagons.

Our earliest analysis with RA was synchronic rather than diachronic, using only a single year. It showed that most of the information in the data is captured by the neighbors directly north, west, east, and south, thus allowing the diagonal neighbors to be ignored [14]. In the field of Cellular Automata, this four-cell neighborhood is known as the von Neumann Neighborhood (VNN), while the full set of eight neighbors is the Moore Neighborhood (Figure 6). Hence, we have used the VNN for all of the subsequent experiments. To summarize the results from prior work, we found that for the geographic region being examined, the VNN captured 88% of the information in the data, also showing a strong southern influence on the DV (central cell). For example, one finding of this earlier study was that if the southern cell in the VNN was Grasses, regardless of what the north, east, and west cells were, the probability that the DV would also be Grasses was 57%. These initial results were promising enough to undertake the multi-year Evergreen Forest study reported in this paper.

## 2. Materials and Methods

NLCD data were acquired from the USGS website, along with the results of the change analysis for each land cover type carried out on a per county basis. The study area was defined as the main part of the Willamette Valley in Oregon (Figure 2). This area was chosen for its diversity of land use and proximity to the researchers. The USGS change analysis tool, available on their website, reports results on a county level. In Lane County, which makes up the bulk of the study area, it was clear that the most actively changing categories were Evergreen Forest (EFO), Shrubs (SHBs), and Grassland (GRS) (Figure 4). Five time periods were acquired: 2001, 2006, 2016, and 2021. Extra time slices are available; however, choosing every five years is a way to make the problem more tractable, by limiting the number of variables we need to analyze, and an evenly spaced time step may allow for better modeling results.

Using some custom programming in Python 3.8, we wrote an algorithm that creates a space–time kernel that moves across the NLCD rasters and extracts the north, west, east, and south values adjacent to the center cell of the kernel from each of the previous years (Figure 3). This produces rows of the following form:

**Z1, N1, W1, E1, S1, Z2, N2, W2, E2, S2, Z3, N3, W3, E3, S3, Z4, N4, W4, E4, S4, Z5**

where Z5 is the DV, and the preceding 20 cells are all of the IVs. This kernel operation is a generalization to spatial data of the procedure of “mask analysis” introduced by Klir [12] for temporal data [15]. Rows, having been formatted for RA, are examined and rows where all cells have the same values—about one-third of the data—are removed, thus focusing only on the data that change. Table 1 is a breakdown of the steps for processing these data. 

Because of the number of records produced by the space–time kernel algorithm over this size of study area (~6.7 million rows), we use random resampling methods to produce smaller files of 500K records for training and testing purposes. Rows are further filtered to only select rows which have an EFO occurring somewhere in the row or neighborhood. This filtering establishes that every cell in a row is actively related to an EFO state at some location, either by being itself an EFO or being adjacent to one. Data are stratified by the DV such that 50% of the rows are 1 (EFO) and 50% are 0 (non-EFO), and then shuffled and randomly split into equal sized train and test sets.

The model is then processed using the OCCAM software. The initial output from OCCAM’s Search function is a set of models that are the best candidates for describing the data (Table 2). These candidate models are generated as follows. Search begins with a starting model, which typically and in the present study is the bottom independence model. OCCAM then searches upwards by examining all “parents” of the starting model whose complexities are incrementally increased. The multiple models thus generated define the next higher level (the starting model is level 0). Accompanying each model is a set of quantitative metrics. Of all models generated at every level, OCCAM retains some best numbers, specified by the Search parameter “width”, where “best” is based on some metric (for example, reduction in DV entropy or ΔBIC). The parents of these best models at level l are generated, after which the best width models at level l + 1 are selected. The number of levels to be explored is specified by the Search parameter “levels”. This procedure is known as a “beam search”. This particular search algorithm used by OCCAM is not an intrinsic feature of RA; other search algorithms could in principle be used but have not yet been implemented in OCCAM.

Models are typically output in descending order from the most complex model considered (which might go all the way to the top, the data) to the bottom reference (the independence model). It is commonplace during this part of the analysis to scan from the bottom of the listing upwards to see how the addition of individual IVs increases the predictive power of the model either by the addition of new separate predicting relations or by interaction effects with predicting IVs that are already present in the model.

OCCAM returns for each model that it evaluates (a) metrics of information captured (or equivalently, absence of error), (b) metrics of complexity, (c) metrics that reflect a tradeoff between information and complexity, and (d) supporting metrics. Metrics of type (a) and (b) monotonically increase with model complexity, but metrics of type (c) are not monotonic. The primary RA metric for information captured is %ΔH(DV), the information-theoretic reduction in Shannon entropy of the DV, analogous for continuous variables to the %variance explained. OCCAM also reports normalized information captured: 0 for independence and 1 for the data where all of the IVs are involved in a high ordinality single predictive relation. These two measures are collinear; entropy reduction is an absolute measure, while normalized information is a relative measure. A model could conceivably capture a large fraction of the information in the data but still reduce the uncertainty of the DV by a small amount; in such cases, the data just do not allow successful prediction of the DV. OCCAM also reports conventional machine learning measures of %Correct for the training and test data sets.

**Table 2:** Partial results from Search output in OCCAM, with models increasing in complexity as ascending in the table in the MODEL column. A good model has high values for all of these metrics. The features are denoted in this paper with a number to indicate the time layer with 1 = 2001, 2 = 2006, 3 = 2011, 4 = 2016. The letters correspond to the cardinal directions. The models mentioned in the discussion below are in **bold**.

ID	MODEL	Level	Inf	%ΔH(DV)	ΔBIC	%C(Data)	%Cover	%C(Test)
13*	**IV:W1Z5:N2Z5:S3Z5:Z4Z5**	**4**	**0.723**	**44.3**	**307057**	**83.3**	**98.9**	**83.4**
12*	IV:N1Z5:E2Z5:W3Z5:Z4Z5	4	0.722	44.3	306721	83.3	98.4	83.3
11*	IV:N1Z5:W2Z5:E3Z5:Z4Z5	4	0.722	44.3	306659	83.2	99.0	83.3
10*	**IV:N2Z5:S3Z5:Z4Z5**	**3**	**0.697**	**42.7**	**295910**	**81.5**	**100.0**	**81.5**
9*	IV:W2Z5:E3Z5:Z4Z5	3	0.694	42.6	294846	81.3	100.0	81.4
8*	IV:E2Z5:W3Z5:Z4Z5	3	0.694	42.5	294692	81.3	100.0	81.4
7*	**IV:N2Z5:Z4Z5**	**2**	**0.638**	**39.1**	**271004**	**75.5**	**100.0**	**75.5**
6*	IV:W2Z5:Z4Z5	2	0.636	39.0	270387	75.6	100.0	75.6
5*	IV:E2Z5:Z4Z5	2	0.634	38.9	269312	75.5	100.0	75.5
4*	**IV:Z4Z5**	**1**	**0.491**	**30.1**	**208753**	**75.8**	**100.0**	**75.8**
3*	IV:Z3Z5	1	0.339	20.8	143831	66.7	100.0	66.7
2*	IV:Z2Z5	1	0.325	19.9	138201	68.1	100.0	68.0
1*	IV:Z5	0	0	0	0	50.0	100.0	50.0

**Table 2** **metrics**:

- Inf is the normalized information captured in the model, namely
  [H(p_ind_) − (p_model_)]/[H(p_ind_) − H(p)].
- %ΔH(DV) is the entropy reduction in the DV which indicates 2021 EFO presence or absence.
- ΔBIC is the difference in BIC from the bottom reference, namely BIC(p_ind_) − BIC(p_model_), where
- BIC = [−2 N ∑_ι_∑ _j_ p(DV_i_, IV_j_) ln p_model_ (DV_i_, IV_j_) ] + ln(N) df, where N is the sample size
- %C(Data) is the %correct in the training data to which the models are fit.
- %Cover is the % of state space of predicting IVs present in the (training) data.
- %C(Test) is the %correct of the model on the test data.

The primary metric for complexity is Δdegrees of freedom, the increase in degrees of freedom of the model from the bottom independence model. Another complexity measure is Search level. (For the models shown in Table 2, where each predicting relation has only a single IV, Δdegrees of freedom is proportional to Search level and so has not been included in the table). Metrics that trade off information and complexity are the Akaike Information Criterion (AIC) and the Bayesian Information Criterion (BIC), both of which are linear combinations of model error and model complexity, and Chi-square *p*-values that assess confidence in model information relative to complexity. BIC is a more conservative metric than AIC, giving model complexity a greater penalty in this integrated goodness measure. Because it is less likely to advocate models that overfit, we generally give BIC more importance than AIC. A valuable supporting metric is %Cover, the fraction of the state space of predicting IVs that the training data includes. This indicates how likely it is that when the model is applied to test data it will need to make predictions for IVs states not encountered in the training data. OCCAM notes for all models reported whether the difference between the model and the reference model of independence is statistically significant, and whether there is also a pathway from independence to the model where every step of increasing complexity is statistically significant. We set the significance threshold for this study at 0.05. The asterisks in the ID column of Table 2 indicate that all the models in this table satisfy these statistical criteria.

When a model is determined to be interesting and accurate enough to examine in detail, it is passed to the Fit function of OCCAM, which produces a report on that specific model using every possible combination of predicting IV states in that model and reporting for each composite IV state the calculated model’s conditional probability of all DV states given the composite IV state, from which OCCAM obtains predictions for the DV in the training and test data and reports statistics on the confidence of these predictions.

See the OCCAM manual [16] for further details about the Search and Fit functions. The results from the Fit analysis are processed in R-Studio and Excel, where it is easy to freeze column values to be constant, see how the other variables behave, and use colors to see patterns. Metrics for each prediction, based on a unique set of IV variable classes, are filtered out so that only the higher frequency combinations of variables are included in the final analysis. Further refinement of the model may be suggested, going back to OCCAM Search, or the Fit results may be used by other methodologies.

## 3. Results

The DV in our OCCAM search is Z5, the state of the center cell at 2021 whose possible values are 1 for code 42 (EFO) and 0 for everything else; this recoding is handled in the OCCAM input file variable bloc header. The IVs are the Center cells from the previous years (Z1, Z2, Z3, Z4) and the north, west, east, and south neighbors of the center cells in 2001 through 2016, namely the set of 20 (IV) variables:

**{Z1, N1, W1, E1, S1, Z2, N2, W2, E2, S2, Z3, N3, W3, E3, S3, Z4, N4, W4, E4, S4}**

As noted above, models obtained by an OCCAM search that allows loops include multiple predictive relations. The result of such a search with parameters level = 4 and width = 3 is shown in Table 2, with key performance metrics for each model. The results are grouped by level.

Working upwards in Table 2 from the bottom reference model, at each numbered level, the best performing model based on %ΔH(DV) is listed at the top of that group, **in bold**. So, the top of level 1 (model 4) includes the IV predictor Z4 (the center cell at time 4), while at the top of level 2 (model 7), we see that the best *additional* predictor is N2, the north cell from time 2, in combination with Z4. Note that OCCAM did not pick any of the other previous center cells (Z) as the best level 2 additional predictor; instead, more information was gained from the north cell three time periods back.

Continuing up, at level three (model 10) we add S3, the south cell from 2011, such that now we have three predicting IV components producing an accuracy of ~80%. However, at level 4, once the %Cover drops below 100%, we begin to produce erratic results when reproducing the calculations. Each random draw of 1 million rows (500K each for test and train) produces the same results up through level 3 (model 10), but after that they drift slightly.

This result was concerning enough that we ran three different random draws from the total pool of 4 million rows. Examining the results showed that there seems to be some interesting substitutions in how the models are using the information to construct the best model. For example, at level 4 we produce two identical models (B and C), but in the third model (A) we have substitutions in some of the IVs, where S3 and N2 are substituted by S2 and N3 (as shown in Table 3), swapping the north and south locations by one time period. At level 5 (Table 3), we obtain one cell from time 1, usually N1; one from time 2, with no discernible pattern; and one more from time 3. Then, each model contains S3 and Z4. At level 6 (Table 3), we obtain fused IVs from times 1 and 3; one cell from time 2; and one cell from time 3, which will be S3 (unless it is fused with a time 1 element). These results are all accompanied by a drop in %Cover to below 100% (Table 2 shows this for level 4; there are further reductions in %Cover at levels 5 and 6).

Because there was agreement from all of the models up through level 3 (Table 2), the best model to use for making predictions from these data could be chosen as model 10. This model is **N2Z5:S3Z5:Z4Z5**, namely the north cell from 2006, the south cell from 2011, and the center cell from 2016. However, as we just reported, there were nearly identical models at level 4, and the extra time slice that it adds to the model helps us to observe the pattern of landscape development from Grasses to Shrubs to Forest. So, going up one more level, we choose the following as the dominant model for performing OCCAM Fit calculations to predict EFO:

**IV:W1Z5:N2Z5:S3Z5:Z4Z5**

This model has four predicting IVs: the west cell from time 1; the north cell from time 2, the south cell from time 3; and the center cell from time 4. Giving this chosen model to the Fit function lets us examine all combinations of the states of predicting IVs and their resulting conditional DV probability distributions. Table 4 shows a partial listing of the results from Fit, sorted by frequency so that the most frequent combinations (states or rows) are at the top. In Table 4, predictions of the DV are made from model conditional probabilities, not data DV probabilities. One predicts non-EFO or EFO based on whichever model conditional DV probability is greater. Thus, for the IV state on row 1, one predicts non-EFO because 59.2% > 40.8%, and we are 68.8% correct, because that is what the actual data show. The table lists %Correct for the predictions of each IV state, and the %Correct of a *model* prediction will equal the % of the *data* conditional probability of that state.

**Figure 7:**
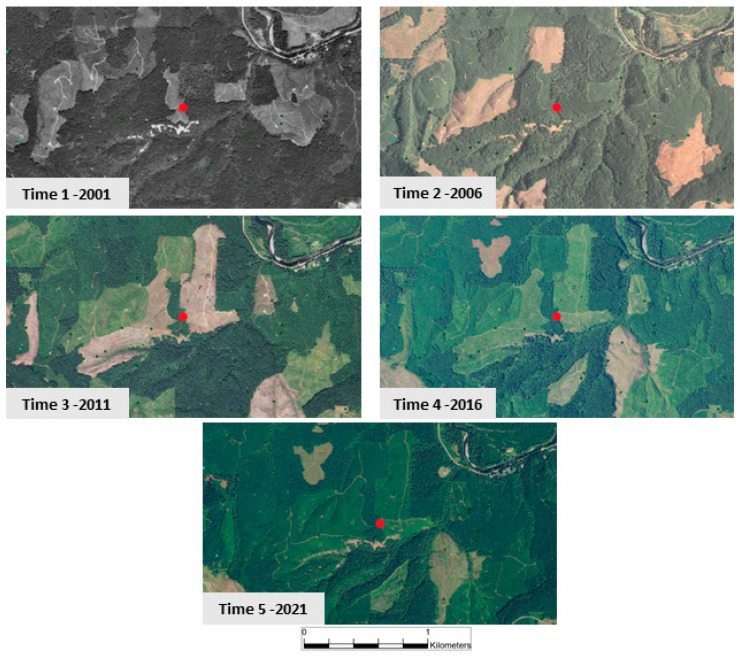
Spatial query of Row 4 from Table 4 results are the red dots, one in the center of each air photo. Panels show the time series of forest patches in various stages of clear cutting cycles, where immature forests are classified as Shrubs, and fresh cuts are Grasses. Several areas appear ready to be harvested in the final panel.

We observe in Table 4 that when an entire space–time neighborhood is in the state of EFO (row 1), there is a 59.2% model probability for the center cell to change to non-EFO. We interpret this as indicating the high likelihood of forest harvesting. Further down the table, there are various combinations of EFO, SHB (Shrubs), and GRS (Grasses), several of which also lead to forest retention or gain. Row 5 is interesting because it has all cells in SHB but predicts forest gain. We interpret this as indicating forest regrowth after harvesting and after an initial phase of Grasses. Row 4 is an interesting combination of cells that has a nearly 100% prediction for forest retention and shows the pattern of GRS -> SHB -> EFO, with this pattern also being clearly visible in Row 7. Row 4 is pulled out for examination of the maps behind this in Figure 7. Figure 7 illustrates the cycles of clear-cut practices and regrowth, as various patches are harvested and then grow back. The dynamic nature of the EFO data element from Figure 4 is clearly displayed in this landscape.

Figure 7 consists of aerial photography of the same geographic area for the years 2001 through 2021, the time period of the data analysis. The first period was only available in black and white, while subsequent years are in full color. The darkness of green in a particular patch is relative to the forest maturity, with darker colors indicating more mature sections that are potentially subject to harvest.

## 4. Discussion

We have shown that Reconstructability Analysis, an information-theoretic modeling methodology, can extract useful results from a spatial data set in its native categorical format without an encoding conversion. In machine learning, this is atypical. RA can characterize and help explain spatial dynamics. By examining the models from OCCAM, we are able to determine which variables contribute the most to prediction of the state of the dependent variable, and these can be used for future predictions. From the Section 3, we observe that the model is capturing a pattern of landscape evolution that roughly follows the pattern of EFO -> GRS -> SHB -> EFO. Often, EFO is followed by GRS, which we interpret as a clear cut. The number of time periods spent in each state varies; sometimes, there are two time periods of SHB before EFO and sometimes there are one or as many as four. Usually, there is one period of GRS followed by SHB, though there is one exception. There are some interesting patterns in rows 11 and 12 (Table 4) suggesting some symmetry and also that there may be a shorter pattern, perhaps Christmas Tree farms or something similar happening on a smaller time frame. It is general knowledge in the Pacific northwest USA that many forests are on a harvest cycle, and this appears to be captured in this current analysis.

**A summary of the key findings:**

- Prediction of the 2021 Evergreen Forest state is ~80% accurate on test data using information from a subset of the previous years 2001 through 2016 (model 10, Table 2).
- This accuracy is obtained from a model that predicts by using the states of a specific combination of neighbors from 2001 through 2016 and the center cell from 2016. The set of neighbors for prediction were obtained by OCCAM’s Search function.
- The main finding from the analysis is that clear-cut practices are the reason that we see such dynamics in the EFO, SHB, and GRS classes from Figure 3.
- The actionable finding is that we could use these patterns to preserve forests that are nearing their harvest date. By intersecting GIS layers of land ownership with forests that are in the state of row 1 from Table 4 (all EFO), contact could be made with owners to see if preservation is an option. This could be useful under climate stress to keep wildlife corridors connected, with the additional potential for carbon sequestration.

Spatial autocorrelation is a well-known phenomenon, in which data that are near each other tend to be more similar than those that are farther apart. This study explores this aspect of NLCD GIS data. Several results from this analysis are surprising. One is that rows that are all in the same EFO state have a higher probability of changing to a non-Evergreen Forest state than those that have a single non-EFO cell in the row. The opposite is also true; when all of the predicting cells are Shrubs, the most likely outcome is to change to EFO.

The NLCD data are highly dynamic, varying over a wide range of possible outcomes over a broad and diverse spatial area. Further examination of these data in more detail, breaking out different classes for example, and testing our methodology in different habitats could suggest other actionable implications. For example, looking at the predictors for the creation of shrubland could assist in habitat preservation for the Sage Grouse and other species that rely on this environment in the high deserts of Eastern Oregon (USA).

Predicting when specific patches of Evergreen Forest are nearing their harvest date and overlaying that information with land ownership maps can give preservationists a chance to intervene before the clear-cut practice. Focusing on individual NLCD classes, as we did with Evergreen Forests, allows for the detection of conditions on the ground that may need attention. This more granular analysis, in conjunction with domain experts from the field of land use change, should yield even more interesting findings. We invite collaboration.

Real-time satellite data are constantly being acquired, and by comparing them to specific past data we may improve our understanding of what is occurring. This technique for analyzing satellite data has possible implications for monitoring and predicting land use changes during the climate change crisis the planet is experiencing. For example, if we know that a certain combination of past states plus a specific current state leads to the loss of a particular habitat, we can set up a monitoring script that analyzes real-time data from satellites and run an algorithm based on OCCAM’s results. These could be run for 1000s of scenarios, as computing power is no longer an issue. By monitoring recent data with these algorithms, critical habitat loss can be prevented or mitigated, and land use planning can be improved.

OCCAM and the RA techniques based on information theory are shown to be capable of extracting useful information from categorical raster data, as illustrated in this initial spatial–RA adventure. Further work is planned, using different Dependent Variables and research questions, such as landslide susceptibility and wildfire intensity prediction. A planned implementation of RA as a Python module which will be publicly available (Occam-RA GitHub site 2024 [17]) is also currently underway.

## Figures and Tables

**Table 1:** Data flow for raster data analysis with Reconstructability Analysis (OCCAM). Extraction was conducted using ArcPro 3.3 GIS software, with further processing in Python.

Processing Step	Results
Extract rows that include the DV (center cell Z5) and space–time VNN neighborhood at times 1 through 4 at every location in the study area	~11 million rows of 20 variables generated
Eliminate rows that have all uniform values	~6.7 million rows retained
Select rows that have Evergreen Forest (NLCD code 42) anywhere in the row	~4 million rows retained
Stratify data so that ½ are EFO present ½ are EFO absent, shuffle, and split into train/test sets	500K rows in each train/test set, replicated 3 times
Add headers for OCCAM input file, reclassifying 15 to 5 classes with rebinning code in the variable block, also recoding Z5 to 1 for code 42, and 0 for all other values	Classes collapsed to: Water, Developed, and Agriculture, Shrubs, Grasses, Mixed/Deciduous Forest, Evergreen Forest
Upload data to OCCAM and run Search	Report generated
Select best model from Search and run Fit on it	Report generated
Extract model predictions from Fit output and analyze with R-Studio 2024, and Excel 2021	Final results

**Table 3:** Top models from each level showing substitutions between cardinal directions and time layers. Each 500K training draw is from a population of 4 million, with an accompanying 500K test set, randomized before split. All three data sets agreed on the top models for levels 1 through 3. Level 4 substitutions shown in red.

Level	500K-A	500K-B	500K-C
**6**	**IV:N1E3Z5:W2Z5:S3Z5:Z4Z5**	**IV:W1S3Z5:N2Z5:E3Z5:Z4Z5**	**IV:N1E3Z5:W2Z5:S3Z5:Z4Z5**
**5**	**IV:N1Z5:W2Z5:E3Z5:S3Z5:Z4Z5**	**IV:W1Z5:N2Z5:E3Z5:S3Z5:Z4Z5**	**IV:N1Z5:E2Z5:W3Z5:S3Z5:Z4Z5**
**4**	**IV:W1Z5:S2Z5:N3Z5:Z4Z5**	**IV:W1Z5:N2Z5:S3Z5:Z4Z5**	**IV:W1Z5:N2Z5:S3Z5:Z4Z5**

**Table 4:** Partial output from the Fit action of the OCCAM program, with each unique combination of IVs listed along with the conditional DV probabilities (expressed in %) and %C (% Correct of the training data, which are nearly identical to the results from the test data) of the model prediction for each IV. Data are sorted in reverse order based on the count of the data in the column frequency. Evergreen Forests are coded as EFOs, Shrubs are SHBs, and Grasses are GRSs, Mixed and Other Forests are MFOs. Water, Developed, and Agriculture (WDA) are all grouped into one class consisting of HID, MID, LID, WTR, EHW, WDW, CRP, and PAS, but this category and MFO do not appear in this abbreviated table.

	Times 1 through 4					Time 5				
	IVs					DV (Data)		DV (Model)		
Row	W1	N2	S3	Z4	Frequency	% Non-EFO	% EFO	% Non-EFO	% EFO	%C
1	**EFO**	**EFO**	**EFO**	**EFO**	90363	68.8	31.2	59.2	40.8	68.8
2	**EFO**	**EFO**	**EFO**	**GRS**	28489	95.4	4.6	98.1	1.9	95.4
3	**EFO**	**EFO**	**GRS**	**SHB**	26066	42.4	57.6	47.2	52.8	57.6
4	**GRS**	**SHB**	**SHB**	**EFO**	14235	2.8	97.2	2.9	97.1	97.2
5	**SHB**	**SHB**	**SHB**	**SHB**	13144	19.1	80.9	9.2	90.8	80.9
6	**EFO**	**EFO**	**EFO**	**SHB**	12886	71.4	28.6	77.7	22.3	71.4
7	**EFO**	**GRS**	**GRS**	**SHB**	12650	21.6	78.4	18.5	81.5	78.4
8	**SHB**	**SHB**	**EFO**	**EFO**	11668	7.5	92.5	12.2	87.8	92.5
9	**SHB**	**SHB**	**SHB**	**EFO**	10919	5.4	94.6	4.1	95.9	94.6
10	**EFO**	**GRS**	**SHB**	**SHB**	9047	15.7	84.3	21.2	78.8	84.3
11	**EFO**	**EFO**	**SHB**	**EFO**	8472	18.6	81.4	30.6	69.4	81.4
12	**EFO**	**SHB**	**EFO**	**EFO**	8344	14.8	85.2	27	73	85.2
13	**EFO**	**GRS**	**SHB**	**EFO**	7816	8.6	91.4	10.1	89.9	91.4

## Data Availability

National Land Cover Data are available for download, and the Python code for processing into RA tabular format is in the Supplementary Materials.

## Supplementary Materials

The following supporting information can be downloaded at: https://github.com/percyd/occam (accessed on 12 June 2024), a Python notebook with code for extracting and processing data from NLCD for use in OCCAM.
